# Transient Receptor Potential Ankyrin 1 (TRPA1) Mediates Hydrogen Sulfide-induced Ca^2+^ Entry and Nitric Oxide Production in Human Cerebrovascular Endothelium

**DOI:** 10.2174/011570159X349872250124124612

**Published:** 2025-02-13

**Authors:** Teresa Soda, Valentina Brunetti, Giovambattista De Sarro, Gerardo Biella, Francesco Moccia, Roberto Berra-Romani, Giorgia Scarpellino

**Affiliations:** 1 Department of Health Science, University Magna Graecia of Catanzaro, 88100 Catanzaro, Italy;; 2 Department of Biology and Biotechnology, Laboratory of General Physiology, “L. Spallanzani”, University of Pavia, 27100 Pavia, Italy;; 3 Department of Medicine and Health Sciences “V. Tiberio”, University of Molise, 86100 Campobasso, Italy;; 4 Department of Biomedicine, School of Medicine, Benemérita Universidad Autónoma de Puebla, 72410 Puebla, Mexico

**Keywords:** Hydrogen sulfide, TRPA1, Ca^2+^ signaling, nitric oxide, hCMEC/D3 cells, cystathionine γ-lyase, ATP

## Abstract

**Introduction:**

The gasotransmitter hydrogen sulfide (H_2_S) modulates various brain functions, including neuron excitability, synaptic plasticity, and Ca^2+^ dynamics. Furthermore, H_2_S may stimulate nitric oxide (NO) release from cerebrovascular endothelial cells, thereby regulating NO-dependent endothelial functions, such as angiogenesis, vasorelaxation, and cerebral blood flow (CBF). However, the signaling pathway by which H_2_S induces NO release from cerebrovascular endothelial cells is still unclear.

**Methods:**

Herein, we exploited single-cell imaging of intracellular Ca^2+^, H_2_S, and NO levels to assess how H_2_S induces Ca^2+^-dependent NO release from the human cerebrovascular endothelial cell line, hCMEC/D3.

**Results:**

Administration of the H_2_S donor, sodium hydrosulfide (NaHS), induced a dose-dependent increase in (Ca^2+^)_i_ only in the presence of extracellular Ca^2+^. NaHS-induced extracellular Ca^2+^ entry was mediated by the Ca^2+^-permeable TRPA1 channel, as shown by pharmacological and genetic manipulation of the TRPA1 protein. Furthermore, NaHS-dependent TRPA1 activation led to NO release that was abolished by buffering the concomitant increase in (Ca^2+^)_i_ and inhibiting eNOS. Furthermore, the endothelial agonist, adenosine trisphosphate (ATP), caused a long-lasting elevation in (Ca^2+^)_i_ that was driven by cystathionine γ-lyase (CSE)-dependent H_2_S production and by TRPA1 activation. Consistent with this, ATP-induced NO release was strongly reduced either by blocking CSE or by inhibiting TRPA1.

**Conclusion:**

These findings demonstrate for the time that H_2_S stimulates TRPA1 to induce NO production in human brain microvascular endothelial cells. Additionally, they show that this signaling pathway can be recruited by an endothelial agonist to modulate NO-dependent events at the human neurovascular unit.

## 1 INTRODUCTION

The gasotransmitter nitric oxide (NO) is recognized to be a key regulator of neurovascular coupling (NVC), *i.e*., the mechanism in which an elevation in neuronal activity leads to an elevation in local cerebral blood flow (CBF), in human brain circulation [1-3]. *In vivo* studies conducted on brain microcirculation have shown that microvascular endothelial cells that are located at the post-arteriole transitional zone of the capillary bed release NO, thereby tuning CBF within downstream capillaries, in response to neuronal activity [4-6]. Neuronal activity stimulates G_q_ protein-coupled receptors (G_q_PCRs) on nearby endothelial cells, which leads to the stimulation of Ca^2+^ mobilization from the endoplasmic reticulum (ER) through inositol-1,4,5-trisphosphate (InsP_3_) receptors (InsP_3_Rs), resulting in the Ca^2+^-dependent activation of the endothelial NO synthase (eNOS) [4, 6]. Consistent with this, the genetic modulation of eNOS expression has been shown to severely impair NVC in different transgenic mouse models [7, 8]. Parallel studies confirmed that several neurotransmitters and modulators, including acetylcholine [9], glutamate [10], γ-aminobutyric acid (GABA) [11], and histamine [12], stimulate eNOS activity by inducing an elevation in (Ca^2+^)_i_ in cultured human brain microvascular endothelial cells [13, 14].

Hydrogen sulfide (H_2_S) is recognized as the third member of the gasotransmitter family, which also includes carbon monoxide [15-20]. H_2_S modulates a growing number of brain functions [17, 20], including neuron excitability [21], synaptic plasticity [22, 23], vascular tone, and CBF [20, 24, 25]. In the brain, H_2_S is generated from L-cysteine by three major enzyme systems, such as cystathionine β-synthase (CBS), 3-mercaptopyruvate sulfurtransferase (3-MST), and cystathionine γ-lyase (CSE) [17, 20, 26]. Cerebral microvessels express both CSE and CBS [20, 27, 28], but CSE is regarded as the major source of the endothelium-derived H_2_S that induces vasodilation and increases local CBF [20, 26, 27, 29]. Depending on the brain region and the species, H_2_S can induce vasodilation in brain microcirculation by activating a variety of K^+^ channels or by inhibiting voltage-gated L-type Ca^2+^ channels in vascular smooth muscle cells (VSMCs) [20, 27]. Furthermore, H_2_S can induce vasorelaxation in rat pial arteries by inhibiting the RhoA-Rho-kinase (ROCK) signaling pathway, thereby reducing the phosphorylation of myosin light chain (MLC) and causing VSMC relaxation [30]. Finally, H_2_S can also increase CBF by stimulating endothelium-dependent NO release, thereby activating the protein kinase G (PKG)-dependent vasorelaxing pathway in VSMCs [25]. H_2_S can stimulate NO release from vascular endothelial cells, thereby regulating many NO-dependent endothelial functions, including angiogenesis and vasorelaxation [16, 19, 20, 31]. The eNOS is a Ca^2+^-dependent enzyme [4, 6], which can be activated by H_2_S *via* induction of phosphatidylinositol 3-kinase (PI3K)/Akt signaling and Akt-dependent eNOS phosphorylation. Surprisingly, only a little information is available regarding the mechanisms by which H_2_S induces Ca^2+^-dependent eNOS activation in vascular endothelial cells [32]. H_2_S can increase the endothelial (Ca^2+^)_i_ by releasing ER Ca^2+^ through InsP_3_Rs [33] and/or ryanodine receptors (RyRs) [32, 34]. H_2_S also induces extracellular Ca^2+^ inflow across the plasma membrane [35], but the endothelial Ca^2+^ entry pathway targeted by H_2_S has not yet been carefully investigated [36, 37]. However, H_2_S can modulate several members of the Transient Receptor Potential (TRP) superfamily of non-selective cation channels, including TRP Ankyrin 1 (TRPA1) [38, 39] and TRP Vanilloid 1 (TRPV1) [40, 41]. Understanding the mechanisms by which H_2_S stimulates Ca^2+^-dependent eNOS activation will therefore shed light on a potentially critical vasorelaxing signaling pathway at the neurovascular unit (NVU). In addition, it could highlight a yet unexplored molecular target for the design of novel neuroprotective strategies for neurological disorders, such as brain ischemia [25] or traumatic brain injury.

The hCMEC/D3 cell line is widely employed as an *in vitro* model of human brain microvascular endothelium [42-45] and is currently exploited to elucidate the functional roles and underlying mechanisms of endothelial Ca^2+^ signaling at the human NVU [11, 46-49]. Additionally, hCMEC/D3 cells express both TRPV1 [48] and TRPA1 [49]. Herein, we described for the first time the mechanisms by which H_2_S induces intracellular Ca^2+^ signals and NO release in hCMEC/D3 cells. We found that the administration of the H_2_S donor, sodium hydrosulfide (NaHS), induced a dose-dependent elevation in (Ca^2+^)_i_ only in the presence of extracellular Ca^2+^. NaHS-induced extracellular Ca^2+^ entry was mediated by the Ca^2+^-permeable TRPA1 channel and led to robust NO release, which was abolished by inhibiting the concomitant increase in (Ca^2+^)_i_ and eNOS activity. Furthermore, the endothelial autacoid, adenosine trisphosphate (ATP), caused a long-lasting elevation in (Ca^2+^)_i_ that was driven by CSE-dependent H_2_S production and by TRPA1 activation. Consistent with this, ATP-induced NO production was strongly decreased either by blocking CSE or by inhibiting TRPA1. These findings demonstrate for the first time that H_2_S stimulates TRPA1 to induce NO production in human cerebrovascular endothelial cells. In addition, they show that this signaling pathway can be recruited by an endothelial agonist to modulate NO-dependent events at the human NVU.

## 2 MATERIALS AND METHODS

### 2.1 Cell Culture

Human cerebral microvascular endothelial cells (hCMEC/D3) were cultured as described in [11, 49]. The cells were grown in tissue culture flasks coated with 0.1 mg/mL rat tail Collagen type 1, and containing the EBM-2 medium (Lonza, Basel, Switzerland) supplemented with 5% fetal bovine serum, 1% Penicillin-Streptomycin, 5 μg/mL ascorbic acid, 1.4 μM hydrocortisone, 1/100 chemically defined lipid concentrate (Life Technologies, Milan, Italy), 10 mM HEPES and 1 ng/mL basic fibroblast growth factor. The flasks were then placed in a cell culture incubator in which they were maintained at 37°C, 5% CO_2_ saturated humidity. For imaging recordings, we used hCMEC/D3 cells between passages 25 and 35. Cell lines were purchased from Cedarlane Labs.

### 2.2 Solutions

For imaging recordings, the cells were bathed with a Physiological Salt Solution (PSS) having the following composition (in mM): 150 NaCl, 6 KCl, 1.5 CaCl_2_, 1 MgCl_2_, 10 Glucose, 10 Hepes. In the Ca^2+^-free solution (0Ca^2+^), Ca^2+^ was replaced with 2 mM NaCl, and 0.5 mM EGTA was added. Solutions were titrated to pH 7.4 with NaOH. The osmolality of PSS was measured with an osmometer (Wescor 5500, Logan, UT, USA) and was equal to 300-310 mOsm/L.

### 2.3 (Ca^2+^)_i_, NO, and H_2_S Imaging

Cells were seeded on glass coverslips coated with gelatin at a density of 5,000 cells/cm^2^ for 24-48 hours [50]. Subsequently, cells were loaded the Ca^2+^-sensitive fluorophore Fura-2 acetoxymethyl ester (2 µM Fura-2/AM) in PSS for 30 minutes at 37°C and 5% CO_2_, as described in [11, 49]. At the end of the incubation period, the coverslip was first washed in PSS and then attached to the bottom of a Petri dish, which was placed under an upright epifluorescence Axiolab microscope (Carl Zeiss, Oberkochen, Germany) and observed with a Zeiss ×40 Achroplan objective [11, 49]. The ratiometric signal emitted by Fura-2 was measured by alternately exciting the dye at 340 and 380 nm with a filter wheel (Lambda 10, Sutter Instrument, Novato, CA, USA) and detecting the emitted light at 510 nm. Single-cell imaging was performed by drawing from 10 up to 40 “regions of interest” (ROIs) around isolated hCMEC/D3 cells. Changes in (Ca^2+^)_i_ was evaluated by measuring the ratio of the fluorescence intensity emitted at 510 nm when excited at 340 nm and 380 nm (F_340_/F_380_) for each ROI. An elevation in (Ca^2+^)_i_ produces an elevation in the ratio [11, 49]. The sampling rate was 1 measurement every 3 seconds. The experiments were carried out at room temperature (23°C).

NO production was measured by loading hCMEC/D3 cells with 4-Amino-5-methylamino-2′,7′-difluorofluorescein diacetate (1 µM, DAF-FM DA) for 60 minutes in PSS at 22°C [11, 49]. The same imaging set-up described above was used to measure the changes in DAF-FM DA fluorescence but a different filter set, *i.e*., excitation at 480 nm and emission at 535 nm wavelength, was selected. The sampling rate was 1 measurement every 5 seconds and the recordings were carried out at 23°C. The cellular production of NO (NOi) was reported as relative fluorescence (F_535_/F_0_) of DAF-FM DA: F_0_ is the basal fluorescence intensity and F_535_ is the fluorescence intensity obtained during recordings.

H_2_S release was measured by loading hCMEC/D3 cells with SF7-AM (1 μM) for 30 minutes in PSS at 22°C and by using the same imaging setup described above but with a different filter set, *i.e*., excitation at 480 nm and emission at 535 nm wavelength. The emission intensity was denoted as the Relative H_2_S level. The sampling rate was 1 measurement every 5 seconds and the recordings were carried out at 23°C.

### 2.4 Immunoblotting

The expression of the TRPA1 protein was evaluated by immunoblot. Cells grown to 70-80% confluency were washed twice in ice-cold Phosphate Buffered Saline (PBS), scraped with the scraped with RIPA buffer (Pierce^®^ RIPA Buffer, Thermo Fisher Scientific, Waltham, MA, USA), and treated with a protease inhibitor cocktail (Halt™ Protease Inhibitor Cocktail, 1:100, Thermo Fisher Scientific, Waltham, MA, USA). After being vortexed and kept in ice for ten minutes, the lysates were centrifuged at 13,000× g for fifteen minutes at 4°C. Bicinchoninic Acid (BCA) kit (Merck KGaA, Darmstadt, Germany) was used to measure the protein concentrations in the lysates, as indicated by the manufacturer. Following a 30-minute heating period at 37°C, 20 micrograms of lysates were reconstituted in SDS loading buffer and separated on 4-15% Mini-PROTEAN TGX Precast Protein Gels Bio-Rad (Bio-Rad, Hercules, CA; USA). Next, using the Trans-Blot Turbo Transfer device (BioRad, Hercules, CA; USA), the proteins were moved from the gel onto the PVDF Membrane (Trans-Blot Turbo Transfer Pack, Bio-Rad, Hercules, CA; USA). The membranes were blocked by agitating them overnight at 4°C with rabbit anti-TRPA1 (Thermo Fisher Scientific #PA146159 in TBST 5% BSA; 1:500) and anti-β-Actin-Peroxidase (#A385416, 1:1000 in TBST 5% BSA 0.02% sodium azide; Merck KGaA, Darmstadt, Germany) antibodies after they had been incubated for 1 hour at room temperature in TBST (20 mM Tris, 150 mM NaCl, 0.1% Tween 20, pH 7.6) 5% BSA solution. The proper HPR-conjugated antibody (anti-rabbit HRP #31460, 1:2,000 in TBST 5% BSA; Thermo Fisher Scientific, Waltham, MA, USA) was then added to the membranes after they had been washed with TBST. Protein expression differences were assessed using Fiji (ImageJ software).

### 2.5 Gene Silencing

The TRPA1 gene was down-regulated as illustrated in [50, 51]. The Lipofectamine™ RNAiMAX Transfection Reagent [Thermo Fisher Scientific, Waltham, MA, USA] protocol was used to transiently transfect the hCMEC/D3 cells with a specific esiRNA targeting TRPA1(EHU040601, MISSION^®^ esiRNA, 100 nM final concentration) and obtained from Merck (Merck KGaA, Darmstadt, Germany). The transfection was performed by maintaining the cells in Opti-MEM™ I Reduced Serum Medium (Thermo Fisher Scientific, Waltham, MA, USA), as indicated by the manufacturer. The esiRNA-Lipofectamin complex was removed four hours after transfection, and the cells were then placed in new culture media with 5% FBS. The cells were then allowed to grow under the chosen protocol while being maintained in an incubator at 37°C and 5% CO_2_.

### 2.6 Statistics

All the data belong to three independent biological replicates. The difference between the ratio (F_340_/F_380_) at the Ca^2+^ peak and the mean ratio of the 60-second baseline before NaHS application was used to calculate the NaHS-evoked initial Ca^2+^ peak. The difference between the mean ratio of the 60-second baseline and the ratio at 600 seconds after ATP application was used to calculate the amplitude of the plateau phase [52]. The difference between the maximum increase in DAF-FM DA fluorescence and the average of the 60-second baseline before agonist application was used to calculate the amplitude of agonist-induced NO release [49]. The difference between the maximum increase in SF-7 fluorescence and the average of 60-second baseline before agonist application was used to calculate the amplitude of agonist-induced H_2_S release. GraphPad Prism 7 (GraphPad Software, Inc., La Jolla, CA, USA) was used to analyze the data. Each dataset's normal distribution was verified using the preliminary Shapiro-Wilk test. Statistical analysis was then conducted using parametric tests, the student’s t-test, or the one-way ANOVA test, as appropriate. A significant *p*-value was defined as less than 0.05.

### 2.7 Chemicals

Fura-2 AM and DAF-FM DA were obtained from Thermo Fisher Scientific (Waltham, MA, USA). SF7-AM was purchased from Cayman Chemical (AnnArbor, Michigan, USA), BAPTA/AM (#196419) and L-NIO (L-N^5^-1-Iminoethyl ornithine, Dihydrochloride; # 400600) were purchased from Merck Millipore (Burlington, Massachusetts, United States). All other chemicals were of analytical grade and purchased from Sigma Chemical Co. (Milan, Italy).

## 3 RESULTS

### 3.1 NaHS Induced Dose-dependent Ca^2+^ Signals in hCMEC/D3 Cells

**Fig. (1):**
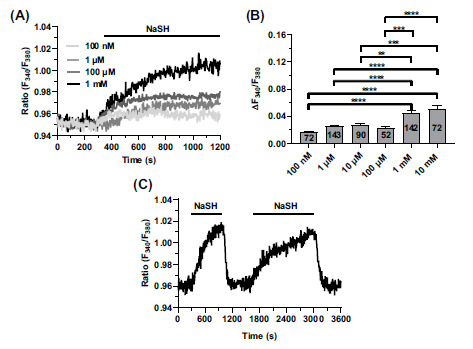
NaHS evokes intracellular Ca^2+^ signals in hCMEC/D3 cells. (**A**) dose-dependent increase in (Ca^2+^)_i_ evoked by NaHS in hCMEC/D3 cells. In this and the following figures, agonists and drugs were administered as indicated by the horizontal bars above the traces. Data are representative of at least three independent experiments. (**B**) concentration-response curve for NaHS-induced increase in (Ca^2+^)_i_ in hCMEC/D3 cells. The data represent the peak increase in fluorescence intensity ratio (340/380 nm) evoked by different concentrations of NaHS. Each point represents the mean ± SEM of at least three independent experiments. **indicates *p <* 0.01, ***indicates *p <* 0.001, ****indicates *p <* 0.0001 (one-way Anova test followed by Turkey post hoc test). (**C**) NaHS (1 mM) evoked a slowly rising increase in (Ca^2+^)_i_. After reaching a plateau, NaHS was removed from the recording solution, thereby causing a rapid decline of the (Ca^2+^)_i_ to the pre-stimulation levels. A second application of NaHS (1 mM) caused a second increase in (Ca^2+^)_i_ that achieved a similar amplitude as the first one.

H_2_S was delivered to hCMEC/D3 cells loaded with the ratiometric Ca^2+^-fluorophore, Fura-2, in the form of NaHS, a water-soluble H_2_S-donating compound, which has long been used to evaluate H_2_S-induced Ca^2+^ signaling in vascular endothelial cells [32-34, 36, 37], as well as other cell types [40, 53]. NaHS induced a dose-dependent increase in (Ca^2+^)_i_ that reached a plateau at 1 mM, (Figs. **1A** and **1B**), while it failed to induce a detectable Ca^2+^ signal at concentrations lower than 100 nM (Fig. **1B**). At each concentration, the Ca^2+^ response to NaHS consisted of a slow increase in Fura-2 fluorescence that occurred immediately after the agonist application and achieved a long-lasting plateau level at ≈700 sec after its onset (Fig. **1A**). Furthermore, the Ca^2+^ response to NaHS quickly decayed to the baseline upon agonist washout from the recording solution (Fig. **1C**). Overall, these findings demonstrate for the first time that the exogenous administration of H_2_S can raise the (Ca^2+^)_i_ in human brain microvascular endothelial cells.

### 3.2 TRPA1-mediated Ca^2+^ Entry is Responsible for NaHS-induced Ca^2+^ Signals in hCMEC/D3 Cells

**Fig. (2):**
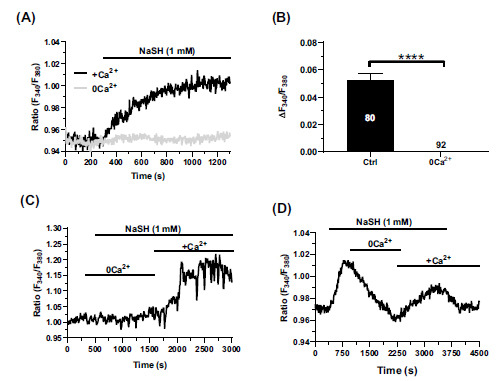
Extracellular Ca^2+^ entry mediates the Ca^2+^ response to NaHS. (**A**) hCMEC/D3 cells were challenged with 1 mM NaHS in the absence of extracellular Ca^2+^ (0Ca^2+^) to assess the role of intracellular Ca^2+^ release and extracellular Ca^2+^ entry in the Ca^2+^ response to NaHS. NaHS (1 mM) evoked an increase in (Ca^2+^)_i_ only in the presence of extracellular Ca^2+^ (+Ca^2+^). (**B**) Mean ± SEM of the peak Ca^2+^ response to NaHS in the presence (+Ca^2+^) and absence (0Ca^2+^) of extracellular Ca^2+^. ****indicates *p <* 0.0001 (Student’s t-test). (**C**) Restoration of extracellular Ca^2+^ (1.5 mM CaCl_2_) after hCMEC/D3 cell stimulation with 1 mM NaHS under 0Ca^2+^ conditions caused a rapid increase in (Ca^2+^)_i_. (**D**) Removal of extracellular Ca^2+^ during the plateau phase of the Ca^2+^ response to NaHS (1 mM) resulted in a slow and reversible decline in (Ca^2+^)_i_.

**Fig. (3):**
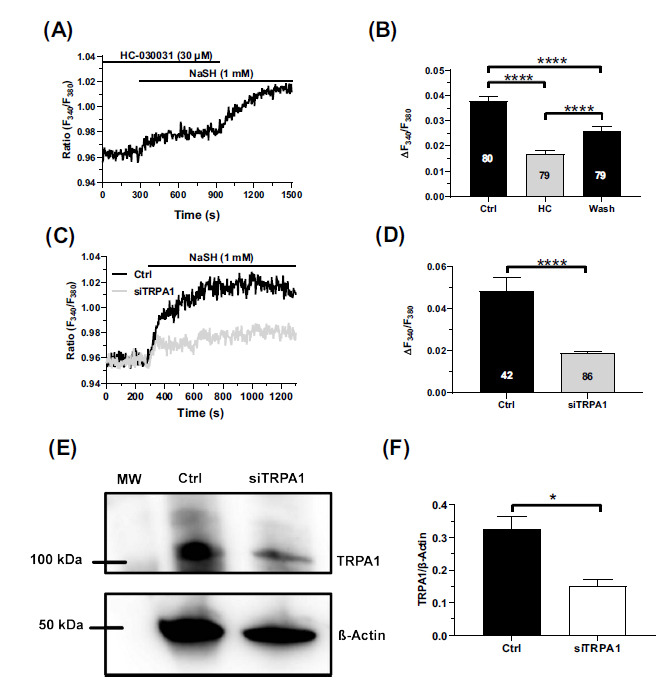
TRPA1 mediates NaHS-evoked extracellular Ca^2+^ entry. (**A**) Representative trace of the small Ca^2+^ response to NaHS (1 mM) in the presence of HC-030031 (30 µM, 30 min), a selective TRPA1 inhibitor. Removal of the inhibitor led to a further increase in (Ca^2+^)_i_, thereby suggesting that TRPA1 mediates NaHS-evoked Ca^2+^ entry in hCMEC/D3 cells. (**B**) Bar histogram depicting the average peak amplitude of the Ca^2+^ response elicited by NaHS (1 mM) in hCMEC/D3 cells in the absence (Ctrl), in the presence of HC-030031 (HC), and after washout of the inhibitor (Wash). Data are represented as mean ± SEM of 3 independent experiments. ****indicates *p <* 0.0001 (one-way Anova test followed by Turkey post hoc test). (**C**) Representative traces of the Ca^2+^ response to NaHS (1 mM) in hCMEC/D3 cells in the absence (Ctrl) or the presence of a siRNA selectively targeting TRPA1 (siTRPA1). The genetic deletion of TRPA1 protein remarkably reduced NaHS-evoked Ca^2+^ entry. (**D**) Bar histogram depicting the average peak amplitude of the Ca^2+^ response elicited by NaHS (1 mM) under the conditions shown in Fig. (**3B**). Data are represented as mean ± SEM of 3 independent experiments. ****indicates *p <* 0.0001 (Student’s t-test). (**E**) Representative Western blot showing TRPA1 protein expression levels in in the absence (Ctrl) or the presence of a siRNA selectively targeting TRPA1 (siTRPA1). β-actin served as a loading control. (**F**) Mean ± SEM of the TRPA1/β-actin ratio expression of 3 independent western blotting experiments. *indicates *p <* 0.05 (Student’s t-test).

To assess whether the Ca^2+^ signal elicited by NaHS was shaped by intracellular Ca^2+^ release, extracellular Ca^2+^ influx, or both, hCMEC/D3 cells were stimulated with 1 mM NaHS in the absence of extracellular Ca^2+^ (0Ca^2+^). NaHS failed to induce an elevation in (Ca^2+^)_i_ under 0Ca^2+^ conditions (Figs. **2A** and **2B**), but the restoration of external Ca^2+^ (1.5 mM CaCl_2_) evoked a rapid Ca^2+^ signal (Fig. **2C**). In addition, when extracellular Ca^2+^ was removed during the plateau phase, the (Ca^2+^)_i_ showed a slow and reversible decline to pre-stimulation levels (Fig. **2D**). Therefore, the exogenous administration of H_2_S is not able to cause intracellular Ca^2+^ release in hCMEC/D3 cells but activates a Ca^2+^-permeable channel on the plasma membrane. A recent study has shown that hCMEC/D3 cells express the H_2_S-sensitive TRPV1 but it does not mediate robust Ca^2+^ signals [48]. The TRPA1 channel protein, which is also sensitive to H_2_S, is robustly expressed in hCMEC/D3 cells, although its function is still unclear [49]. In the presence of the selective TRPA1 inhibitor, HC-030031 (30 µM) [51-55], the Ca^2+^ response to NaHS (1 mM) was significantly (*p <* 0.05) dampened when compared to control conditions (Figs. **3A** and **3B**). Furthermore, NaHS-induced extracellular Ca^2+^ entry was significantly (*p <* 0.05) decreased in hCMEC/D3 cells treated with a small interfering RNA selectively targeting TRPA1 expression (siTRPA1) (Figs. **3C** and **3D**) [51]. The efficacy of TRPA1 deletion by the siTRPA1 employed in this and other investigations [51] was confirmed by immunoblotting analysis (Figs. **3E** and **3F**). Overall, these findings demonstrate that TRPA1 mediates NaHS-induced extracellular Ca^2+^ influx in human brain microvascular endothelial cells.

### 3.3 TRPA1-mediated Ca^2+^ Entry Stimulates NO Release in hCMEC/D3 Cells

**Fig. (4):**
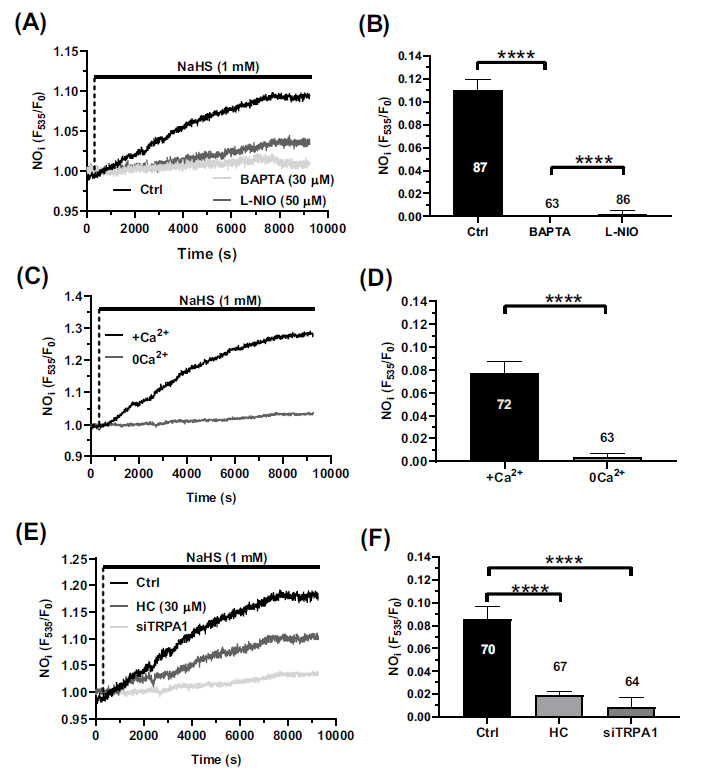
NaHS promotes NO production *via* TRPA1-mediated Ca^2+^ entry. (**A**) hCMEC/D3 cells were loaded with the NO-sensitive fluorophore, DAF-FM DA and stimulated with NaHS (1 mM) in the absence (Ctrl) or presence of BAPTA (30 µM, 2 h), an intracellular Ca^2+^ buffer, or L-NIO (50 µM, 1 h), an eNOS inhibitor. (**B**) Quantification of DAF-FM DA fluorescence intensity in hCMEC/D3 cells treated as in Fig. (**4A**). Data are represented as mean ± SEM of 3 independent experiments. ****indicates *p <* 0.0001 (one-way Anova test followed by Dunnett’s post hoc test). (**C**) NaHS (1 mM) induced NO release in the presence (+Ca^2+^) but not in the absence (0Ca^2+^) of extracellular Ca^2+^. (**D**) Quantification of DAF-FM DA fluorescence intensity in hCMEC/D3 cells treated as in Fig. (**4C**). Data are represented as mean ± SEM of 3 independent experiments. ****indicates *p <* 0.0001 (Student’s t-test). (**E**) NaHS-evoked NO release in the absence (Ctrl) or the presence of a siRNA selectively targeting TRPA1 (siTRPA1) or in the presence of HC-030031 (HC; 30 μM, 30 min). (**F**) quantification of DAF-FM DA fluorescence intensity in hCMEC/D3 cells treated as in Fig. (**4E**). Data are represented as mean ± SEM of 3 independent experiments. ****indicates *p <* 0.0001 (one-way Anova test followed by Dunnett’s post hoc test).

A sustained elevation in (Ca^2+^)_i_ can stimulate NO production *via* eNOS activation in hCMEC/D3 cells [9, 11, 12, 49, 56]. Therefore, we assessed whether the exogenous administration of H_2_S was also able to promote NO production in hCMEC/D3 cells loaded with DAF-FM DA, a NO-sensitive fluorescent probe. One mM NaHS induced a massive increase in DAF-FM DA fluorescence, which was inhibited by BAPTA (30 µM) (Fig. **4A**), a membrane-permeable Ca^2+^ buffer preventing the accumulation of intracellular Ca^2+^, and by L-NIO (50 µM) (Fig. **4A**), which selectively inhibits eNOS activity. The statistical analysis of these results has been shown in Fig. (**4B**). Furthermore, NaHS-induced NO release did not take place under 0Ca^2+^ conditions (Fig. **4C**), as also summarized in Fig. (**4D**). Consistently, the blockade of TRPA1 with HC-030031 (30 µM) (Fig. **4E**) and the genetic suppression of TRPA1 protein expression with the selective siTRPA1 significantly (*p* < 0.05) reduced NaHS-induced NO release (Fig. **4E**). The statistical analysis of these results has been shown in Fig. (**4F**). Taken together, these findings demonstrate that TRPA1-mediated Ca^2+^ entry leads to NO release in human brain microvascular endothelial cells.

### 3.4 TRPA1 Sustains ATP-induced Ca^2+^ Influx and NO Production in hCMEC/D3 Cells

**Fig. (5):**
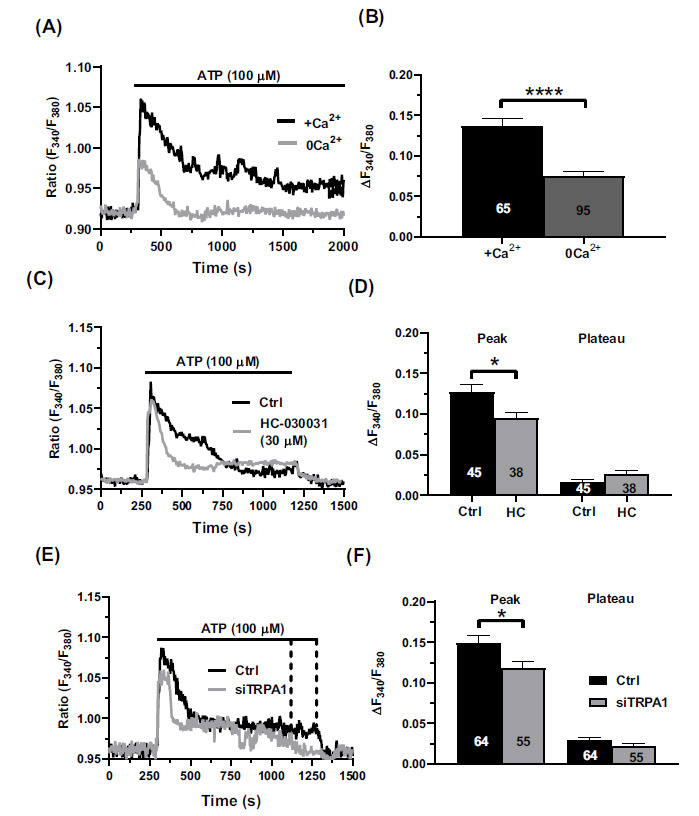
TRPA1 is involved in ATP-induced Ca^2+^ entry. (**A**) In the presence of extracellular Ca^2+^ (+Ca^2+^), ATP (100 µM) caused a biphasic increase in (Ca^2+^)_i_. Under 0Ca^2+^ conditions (0Ca^2+^), ATP (100 µM) evoked a smaller and transient increase in (Ca^2+^)_i_. (**B**) Bar histogram depicting the average peak amplitude of the Ca^2+^ response elicited by ATP in the presence (+Ca^2+^) and absence (0Ca^2+^) of extracellular Ca^2+^. Data are represented as mean ± SEM of 3 independent experiments. ****indicates *p <* 0.0001 (Student’s t-test). (**C**) The pharmacological inhibition of TRPA1 with HC-030031 (30 µM, 30 min) significantly reduced the initial Ca^2+^ peak but did not affect the sustained plateau. (**D**) Bar histogram depicting the average peak amplitude and the average plateau amplitude of the Ca^2+^ response elicited by ATP in the presence (Ctrl) and absence of HC-030031 (HC). Data are represented as mean ± SEM of 3 independent experiments. *indicates *p <* 0.05 (Student’s t-test). (**E**) The genetic suppression of TRPA1 expression with the selective siTRPA1 reduces the initial Ca^2+^ peak without affecting the plateau phase. (**F**) Bar histogram depicting the average peak amplitude and the average plateau amplitude of the Ca^2+^ response elicited by ATP in the presence (Ctrl) and absence of the specific siTRPA1 (siTRPA1). Data are represented as mean ± SEM of 3 independent experiments. * indicates *p <* 0.05 (Student’s t-test).

The findings reported above demonstrate that H_2_S can stimulate TRPA1-mediated Ca^2+^ entry, thereby resulting in NO release in human brain microvascular endothelial cells. Then, we wondered whether this signaling pathway can be recruited by an established endothelial autacoid, such as ATP [46, 57, 58]. Therefore, we first assessed whether TRPA1 supports ATP-induced Ca^2+^ influx in Fura-2-loaded hCMEC/ D3 cells. ATP (100 µM) induced a biphasic increase in (Ca^2+^)_i_, which comprised a rapid Ca^2+^ peak followed that rapidly decayed to a sustained plateau phase (Fig. **5A**). In the absence of extracellular Ca^2+^, ATP evoked a transient elevation in (Ca^2+^)_i_ that showed a lower amplitude and shorter duration as compared to control conditions (Fig. **5B**). These findings confirm that ATP-induced Ca^2+^ signals are initiated by InsP_3_-induced ER Ca^2+^ mobilization, as shown in [46, 57-59], and prolonged by extracellular Ca^2+^ influx. The pharmacological blockade of TRPA1 with HC-030031 (30 µM) (Figs. **5C** and **5D**) and the genetic suppression of TRPA1 protein expression with the selective siTRPA1 (Fig. **5E** and Fig. **5F**) significantly (*p <* 0.05) reduced the initial Ca^2+^ peak but did not affect the sustained plateau. Therefore, TRPA1 supports ATP-induced Ca^2+^ influx during the initial Ca^2+^ peak in human brain microvascular endothelial cells.

### 3.5 CSE Drives ATP-induced H_2_S Production in hCMEC/D3 Cells

**Fig. (6):**
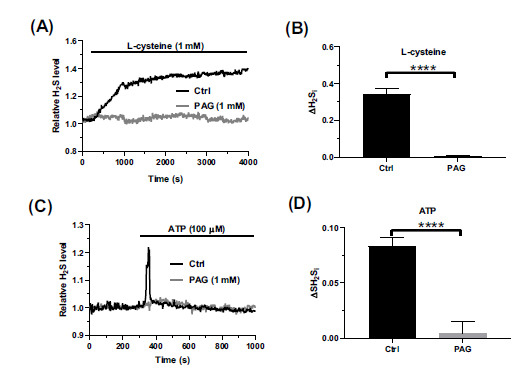
CSE-mediates H_2_S production by L-cysteine and ATP. (**A**) hCMEC/D3 cells were loaded with the H_2_S-selective fluorophore, SF7-AM. The cells were then stimulated with the H_2_S precursor, L-cysteine (1 mM) at the indicated time point. The pharmacological blockade of CSE activity with DL-propargylglycine (PAG; 1 mM, 30 min) abolished L-cysteine-induced H_2_S production. (**B**) Quantification of the peak increase in SF7 fluorescence (ΔH_2_S_i_) induced by L-cysteine in the absence (Ctrl) and presence of PAG (PAG). Data are represented as mean ± SEM of 3 independent experiments. ****indicates *p <* 0.0001 (Student’s t-test). (**C**) hCMEC/D3 cells were loaded with the H_2_S-selective fluorophore, SF7-AM. The cells were then stimulated with ATP (100 µM) at the indicated time point. Representative traces from 3 independent experiments are shown. ATP-induced H_2_S production was inhibited by PAG (1 mM, 30 min). (**D**) quantification of the peak increase in SF7 fluorescence (ΔH_2_S_i_) induced by ATP in the absence (Ctrl) and presence of PAG (PAG). Data are represented as mean ± SEM 3 independent experiments. ****indicates *p <* 0.0001 (Student’s t-test).

Early work has shown that CSE is the main source of H_2_S production in cerebrovascular endothelial cells [20, 26, 27, 29, 30]. L-cysteine is the main substrate for CSE-dependent H_2_S production and has been widely used to evaluate CSE activity in mammalian cells [60, 61]. The endogenous production of H_2_S was tracked in hCMEC/D3 cells loaded with SF7-AM, which has recently been introduced as an H_2_S-selective fluorophore [62, 63]. Preliminary experiments confirmed that the exogenous administration of H_2_S with NaHS (100 µM) caused a sustained increase in the SF7-dependent signal (Fig. **S1**), which is consistent with H_2_S liberation and resembles the kinetics of the concomitant elevation in (Ca^2+^)_i_ (Fig. **1A**). Consistent with this, L-cysteine (1 mM) caused a sustained increase in endogenous H_2_S production that was inhibited by DL-propargylglycine (PAG; 5 mM) (Figs. **6A** and **6B**), which selectively blocks CSE activity [30, 33, 36]. The physiological stimulation with ATP (100 µM) caused an increase in intracellular H_2_S levels that displayed rapid and transient kinetics in 36 out of 62 hCMEC/D3 cells (Fig. **6C**). In the remaining 34 cells, the rapid elevation in H_2_S production preceded a slow wave of H_2_S release that decayed to pre-stimulation levels upon removal of ATP from the perfusate (Fig. **S2**). As for L-cysteine, ATP-induced H_2_S production was inhibited by blocking CSE activity with PAG (5 mM) (Figs. **6C** and **6D**). Therefore, ATP can increase intracellular H_2_S concentration by activating CSE in human cerebrovascular endothelial cells. Furthermore, the rapid kinetics of H_2_S production concur with the aforementioned evidence that TRPA1 supports the initial increase in (Ca^2+^)_i_ but not the following plateau phase.

### 3.6 TRPA1 Sustains ATP-induced NO Release in hCMEC/D3 Cells

**Fig. (7):**
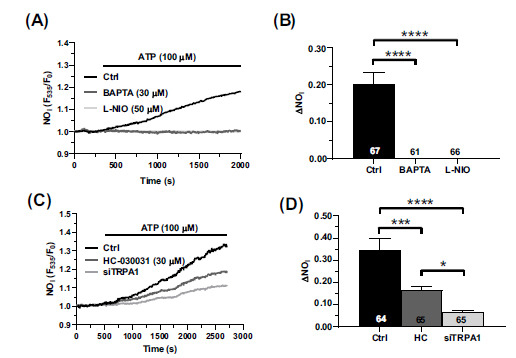
TRPA1 supports ATP-induced NO release. (**A**) hCMEC/D3 cells were loaded with the NO-sensitive fluorophore, DAF-FM DA, and stimulated with ATP (100 μM) in the absence (Ctrl) or absence of BAPTA (30 µM, 2 h) or L-NIO (50 µM, 1 h). (**B**) Quantification of DAF-FM DA fluorescence intensity in hCMEC/D3 cells treated as in Fig. (**7A**). Data are represented as mean ± SEM of 3 independent experiments. ****indicates *p <* 0.0001 (one-way Anova test followed by Dunnett’s post hoc test). (**C**) ATP-evoked NO release in hCMEC/D3 in the absence (Ctrl) or in the presence of the specific siTRPA1 (siTRPA1) or pre-treated with HC-030031 (HC; 30 μM, 30 min). (**D**) quantification of DAF-FM DA fluorescence intensity in hCMEC/D3 cells treated as in Fig. (**7C**). Data are represented as mean ± SEM of 3 independent experiments. ****indicates *p <* 0.0001 (one-way Anova test followed by Dunnett’s post hoc test).

Finally, we investigated whether ATP-elicited elevation in endogenous H_2_S production results in TRPA1-dependent NO release. ATP (100 µM) caused a massive NO release in DAF-FM DA-loaded hCMEC/D3 cells (Fig. **7A**). ATP-induced NO production was suppressed by buffering the (Ca^2+^)_i_ with BAPTA (30 µM; Fig. **7A**) and by interfering with eNOS activity with L-NIO (50 µM; Fig. **7A**). The statistical analysis of these results is shown in Fig. (**7B**). In a subsequent set of experiments, we found that ATP-induced NO release was suppressed by HC-030031 (30 µM; Fig. **7C**), by siTRPML1 (Fig. **7C**), and by PAG (5 mM) (Fig. **7C**). The statistical analysis of these results has been shown in Fig. (**7D**). Therefore, ATP-induced NO release in human cerebrovascular endothelial cells can be supported by CSE activation and the downstream recruitment of the H_2_S-sensitive TRPA1 channel.

## 4 DISCUSSION

The gasotransmitter H_2_S is emerging as a critical regulator of the NVU by activating multiple vasorelaxing pathways, including endothelium-dependent NO release. Herein, we provide the first evidence that the exogenous administration of H_2_S stimulates TRPA1-mediated Ca^2+^ entry in human cerebrovascular endothelial cells, thereby leading to robust NO release. We further show that the endothelial autacoid, ATP, recruits this signaling pathway by causing CSE-dependent H_2_S production and NO release. Therefore, the H_2_S/TRPA1/eNOS signaling pathway can be harnessed by neurotransmitters and neuromodulators to activate NO-dependent functions at the NVU, including vasorelaxation and CBF modulation. These findings provide the molecular substrate to explain the neuroprotective effect afforded by exogenous H_2_S against the ischemia/reperfusion injury in the brain [25].

Single-cell Ca^2+^ imaging recordings revealed that the liberation of exogenous H_2_S from NaHS caused a dose-dependent increase in (Ca^2+^)_i_ that occurred at 100 nM and achieved a plateau at 1 mM. The Ca^2+^ response to NaHS consisted of a slow increase in (Ca^2+^)_i_ that was reversed upon agonist washout. The dose-dependence and the kinetics of NaHS-evoked Ca^2+^ signals are similar to those described in human saphenous vein endothelial cells [34], human dermal microvascular endothelial cells (HMVECs), and breast cancer-derived endothelial cells (B-TECs) [36], while it is different from that described in EA.hy926 cells [33]. In neutral solutions, NaHS dissociates into H_2_S (25%) and HS^-^ (75%). Therefore, the actual concentration of H_2_S that can trigger detectable Ca^2+^ signals in hCMEC/D3 cells ranges between »25 nM and 250 µM. The tissue concentration of free H_2_S in the brain is on the order of 15 nM, but it has been postulated that the intracellular free H_2_S concentration must rise above 100 µM to exert a significant biological effect [35, 64]. The slow dynamics of the H_2_S-dependent increase in (Ca^2+^)_i_ suggests that the underlying Ca^2+^ signaling machinery is the same in endothelial cells from different vascular beds. Nevertheless, the Ca^2+^ response to NaHS is mediated by extracellular Ca^2+^ entry in HMVECs and B-TECs [36], as also shown in rat aortic endothelial cells [37], while it is shaped by ER-dependent Ca^2+^ release through RyRs and/or InsP_3_Rs in human saphenous endothelial cells [34] and EA.hy926 [33]. When NaHS was administered to hCMEC/D3 cells in the absence of extracellular Ca^2+^, it failed to induce a detectable increase in (Ca^2+^)_i_, thereby suggesting that it must gate a Ca^2+^ entry channel on the plasma membrane to evoke a robust Ca^2+^ signal [36, 37, 40]. H_2_S has been shown to gate TRPV1 [40, 41] and TRPA1 [38, 39] by sulfhydration of reactive cysteine residues that are located in their cytosolic moieties [35]. In addition, H_2_S can enhance N-methyl-D-aspartate (NMDA) receptors (NMDARs)-mediated Ca^2+^-permeable currents in rat hippocampus [23]. A recent study showed that NMDARs are expressed in hCMEC/D3 cells but do not mediate sizeable inward currents [10, 13]. However, endothelial NMDARs signal in a flux-independent manner and cause an increase in (Ca^2+^)_i_ by mobilizing the ER and endolysosomal Ca^2+^ pools [10, 13]. Similarly, TRPV1 is weakly expressed and does not mediate detectable Ca^2+^ signals in hCMEC/D3 cells [48]. Therefore, NMDARs and TRPV1 are unlikely to support NaHS-induced Ca^2+^ signals in hCMEC/D3 cells. Conversely, TRPA1 activation by the endogenous agonist, 4-Hydroxynonenal (4-HNE) [51, 65], elicits robust Ca^2+^ signals in hCMEC/D3 cells [49]. Herein, we found that the slow increase in (Ca^2+^)_i_ induced by NaHS was strongly reduced by blocking TRPA1 with the selective inhibitor, HC-030031, and by *TRPA1* gene silencing. Therefore, TRPA1 is the major Ca^2+^ entry pathway that mediates NaHS-induced Ca^2+^ entry in human cerebrovascular endothelial cells. TRPA1 has also been detected in the acidic lysosomal vesicles of dorsal root ganglion neurons [66], but our data strongly suggest that it does not mobilize lysosomal Ca^2+^ under or conditions. hCMEC/D3 cells do not express RyRs, while they express InsP_3_Rs that are seemingly insensitive to NaHS [9]. Future work could explore the possibility that some of the reactive thiol-containing components of the endothelial Ca^2+^ toolkit, such as InsP_3_Rs, are shielded by local H_2_S scavengers, *e.g*. hydroxocobalamin (*e.g*., vitamin B_12a_) [67], and cannot therefore support NaHS-evoked elevation in (Ca^2+^)_i_. This hypothesis has recently been put forward to explain why the same reactive oxygen species activate different components of the endothelial Ca^2+^ machinery in different vascular districts [68].

A global increase in (Ca^2+^)_i_ can lead to eNOS activation and NO release in mouse [4, 6, 14] and human [9, 11, 49, 69] cerebrovascular endothelial cells. Consistent with this, NaHS induced a massive production of NO that was inhibited by blocking either eNOS activity or TRPA1-mediated Ca^2+^ entry. Early work showed that H_2_S was able to induce NO release in a Ca^2+^-dependent manner, but our observations shed novel light on the molecular target, *i.e*., TRPA1, that is activated by H_2_S to recruit eNOS [32]. Of note, TRPA1 could be more efficiently activated by the chemical formation of polysulfides (H_2_S_n_), which are generated by the combination of H_2_S with NO [70]. Therefore, the initial activation of TRPA1-mediated Ca^2+^ entry by H_2_S could be sustained by the production of H_2_S_n_ that follows NO release. TRPA1 activation could provide the mechanism by which H_2_S induces the NO-dependent increase in local CBF [25] and drives NO-dependent angiogenesis in the brain microcirculation [71]. As a consequence, our findings could have implications for the neuroprotective strategies aiming at rescuing CBF through the exogenous administration of H_2_S after the ischemia/reperfusion injury [25, 72]. Under this hypothesis, TRPA1 stimulation has recently been proposed as an alternative approach to rescue CBF in several brain disorders, including brain ischemia [72, 73]. It should be noted that the mechanism by which endothelial TRPA1 channels contribute to regulating CBF in the mouse microcirculation does not involve NO signaling, but the activation of an intercellular Ca^2+^ wave spreading from capillaries to the arteriole-capillary transition zone. Herein, the Ca^2+^ signal is converted into an electrical signal *via* the recruitment of small- and intermediate-conductance Ca^2+^-dependent K^+^ channels (SK_Ca_ and IK_Ca_, respectively), which dilate upstream arterioles by hyperpolarizing their vascular smooth muscle fibers [65, 73]. We cannot rule out the possibility that such a mechanism is also activated by H_2_S in the human brain microvasculature. NaHS-induced Ca^2+^ entry through TRPA1 could also be sensitive to endothelial hyperpolarization [20]. It has long been known that H_2_S may activate ATP-sensitive K^+^ channels, which are likely to be expressed in hCMEC/D3 cells [74], and SK_Ca_ and IK_Ca_ channels, that have recently been detected in hCMEC/D3 cells [49]. The concomitant endothelial hyperpolarization could increase the driving force for Ca^2+^ entry, thereby boosting NaHS-induced Ca^2+^ influx through TRPA1, as shown in rat aortic endothelium [37].

Recent studies suggested that agonist-induced Ca^2+^ signals in vascular endothelial cells could be supported by CSE activation and H_2_S production [36, 37]. ATP is routinely employed to investigate Ca^2+^ dynamics in vascular endothelial cells [46, 57, 58], including hCMEC/D3 cells [46, 59]. Binting *et al*. showed that the G_q_-protein coupled P2Y2 receptor triggers ATP-induced ER Ca^2+^ release through InsP_3_Rs in these cells [46]. Herein, we further showed that the TRPA1 contributes to the initial increase in (Ca^2+^)_i_ induced by ATP. In addition, we demonstrated that ATP also evokes a rapid increase in H_2_S levels that is likely to drive TRPA1 activation during the initial Ca^2+^ peak. The CSE protein is markedly expressed in hCMEC/D3 cells [28] and is regarded as the major source of the endothelium-derived H_2_S in the brain microcirculation [20, 26, 27, 29]. The pharmacological blockade of CSE activity with PAG mimics the effects of TRPA1 inhibition on ATP-induced Ca^2+^ entry and NO release. Therefore, H_2_S is the endogenous agonist by which ATP leads to TRPA1 activation and adds to the list of intracellular messengers, already including 4-HNE, which fulfills this role in cerebrovascular endothelial cells [65, 75]. The mechanism coupling the P2Y2 receptor to CSE activation remains unclear. CSE activity could be enhanced by Akt-dependent phosphorylation or by a triggering increase in (Ca^2+^)_i_ [19, 20]. ATP-induced InsP_3_-dependent ER Ca^2+^ release could rapidly promote CSE activation and initiate H_2_S production in hCMEC/D3 cells. Of note, hCMEC/D3 cells also express CBS and 3-MTS [28]. Nevertheless, the evidence that blocking CSE activity with PAG virtually abolishes H_2_S production induced by both L-cysteine and ATP, strongly suggests that two addition sources of H_2_S play a minor role in H_2_S -dependent Ca^2+^ signals in hCMEC/D3 cells. Further work will have to assess whether H_2_S-dependent TRPA1 activation contributes to the endothelial Ca^2+^ signals induced at the NVU by other agonists, such as acetylcholine and glutamate. Interestingly, the pharmacological and genetic blockade of TRPA1 did not affect the long-lasting plateau phases that follow the initial increase in (Ca^2+^)_i_. This feature is quite interesting as, in a fraction of hCMEC/D3 cells, the production of H_2_S induced by ATP is not transient but continues as long as the agonist is presented to the cells. Identifying the mechanism responsible for the ATP-evoked plateau phase was far beyond the scope of the present investigation. Nevertheless, ATP could activate multiple Ca^2+^ entry pathways in vascular endothelial cells, including P2X receptors [58, 76] and store-operated Ca^2+^ channels (SOCs) [52, 77]. Clearly, in hCMEC/D3 cells showing a transient increase in H_2_S levels, TRPA1 lacks its gating signal, *i.e*., H_2_S, after the initial Ca^2+^ peak and cannot contribute to the plateau phase. On the other hand, in the cells showing a slow wave of H_2_S production after the initial Ca^2+^ peak, either TRPA1 contribution is barely detectable as compared to the other Ca^2+^ entry pathway(s) or it is inhibited despite for the availability of H_2_S. In agreement with this hypothesis, early reports showed that store-dependent and store-independent Ca^2+^ entry routes may not always be activated at the same time [78, 79]. H_2_S has previously been shown to inhibit SOCs [80], but whether store-operated Ca^2+^ entry modulates H_2_S-induced Ca^2+^ signals is still unknown.

## CONCLUSION

The present investigation provides the first demonstration that both exogenous and endogenous H_2_S stimulate TRPA1 to mediate extracellular Ca^2+^ entry and NO release in the human cerebrovascular endothelial cell line, hCMEC/D3. This signaling pathway could be critical to modulating multiple NO-dependent physiological functions, such as CBF and angiogenesis. In addition, it could be exploited to design novel neuroprotective strategies to rescue vascular functions in brain disorders, such as brain ischemia.

## Data Availability

All the data and supporting information is provided within the article.

## LIST OF ABBREVIATIONS

B-TECs: Breast Cancer-derived Endothelial Cells
CBF: Cerebral Blood Flow
ER: Endoplasmic Reticulum
HMVECs: Human Dermal Microvascular Endothelial Cells
MLC: Myosin Light Chain
NO: Nitric Oxide
NVC: Neurovascular Coupling
PBS: Phosphate Buffered Saline
TRP: Transient Receptor Potential
TRPA1: Transient Receptor Potential Ankyrin 1
VSMCs: Vascular Smooth Muscle Cells
