# Supplementary material for: Transient Receptor Potential Ankyrin 1 (TRPA1) Mediates Hydrogen Sulfide-induced Ca2+ Entry and Nitric Oxide Production in Human Cerebrovascular Endothelium
Source: Curr Neuropharmacol. 2025 Feb 13;23(9):1119–33. doi: 10.2174/011570159X349872250124124612 (PMC12272095; doi:10.2174/011570159X349872250124124612)
Supplement: Supplementary file 1 [file CN-23-9-1119_SD1.pdf]

Supplementary Material

Transient Receptor Potential Ankyrin 1 (TRPA1) Mediates Hydrogen Sulfide-induced Ca^{2+} Entry and Nitric Oxide Production in Human Cerebrovascular Endothelium

Teresa Soda[1], Valentina Brunetti[2], Giovambattista De Sarro[1], Gerardo Biella[2], Francesco Moccia[3,#,*], Roberto Berra-Romani[4,#] and Giorgia Scarpellino[2]

[1]Department of Health Science, University Magna Graecia of Catanzaro, 88100 Catanzaro, Italy; [2]Laboratory of General Physiology, Department of Biology and Biotechnology "L. Spallanzani", University of Pavia, 27100 Pavia, Italy; [3]Department of Medicine and Health Sciences "V. Tiberio", University of Molise, 86100 Campobasso, Italy; [4]Department of Biomedicine, School of Medicine, Benemérita Universidad Autónoma de Puebla, 72410 Puebla, Mexico

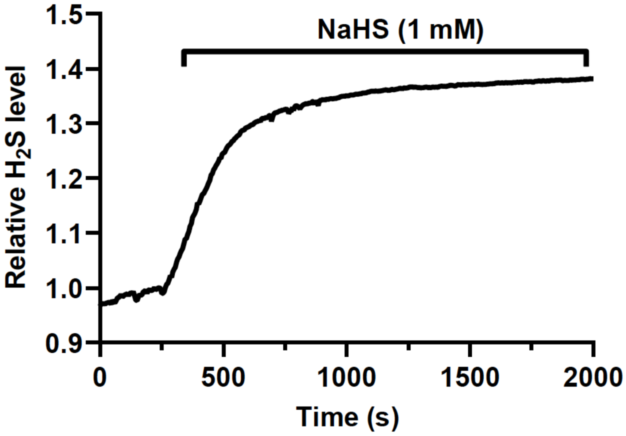

Fig. (S1). SF7-AM is a reliable sensor of H_2S production in hCMEC/D3 cells. The administration of NaHS (100 μM) induced a rapid increase in SF7 fluorescence, which indicates that SF7 is a reliable probe for H_2S production in hCMEC/D3 cells.

1875-6190/25 $65.00+.00 © 2025 Bentham Science Publishers

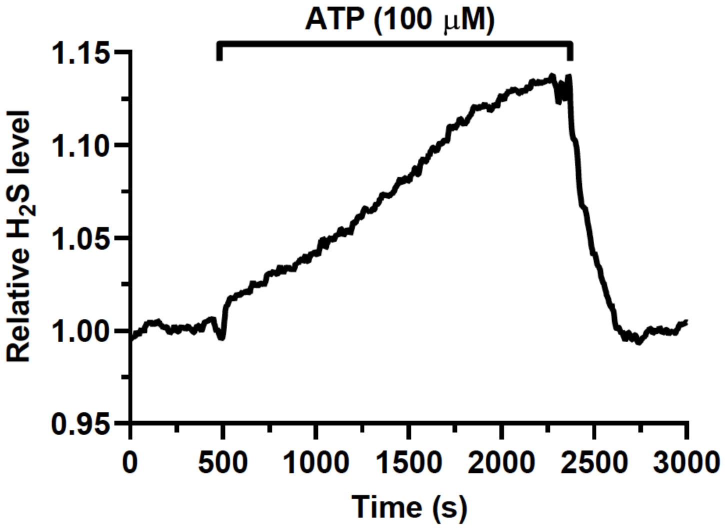

Fig. (S2). Long-lasting pattern of H_2S production induced by ATP. In a fraction of hCMEC/D3 cells, ATP (100 μM) elicited a slow increase in SF7 fluorescence after the rapid initial rise. The production of H_2S decayed to pre-stimulation levels upon ATP removal from the bath.
